# Preparation of Fibrous Three-Dimensional Porous Materials and Their Research Progress in the Field of Stealth Protection

**DOI:** 10.3390/nano14121003

**Published:** 2024-06-09

**Authors:** Peng Zhang, Shuang Zhao, Guobing Chen, Kunfeng Li, Jun Chen, Zhen Zhang, Feiyue Yang, Zichun Yang

**Affiliations:** College of Power Engineering, Naval University of Engineering, Wuhan 430033, China; 21000302@nue.edu.cn (P.Z.); shuangzhao@nudt.edu.cn (S.Z.); chenguob@163.com (G.C.); waws1019@163.com (K.L.); zhangzhen12a@126.com (Z.Z.); feiyuehit@foxmail.com (F.Y.)

**Keywords:** fibrous porous material, aerogel, ceramics, infrared stealth, acoustic stealth, radar stealth

## Abstract

Intelligent and diversified development of modern detection technology greatly affects the battlefield survivability of military targets, especially infrared, acoustic wave, and radar detection expose targets by capturing their unavoidable infrared radiation, acoustic wave, and electromagnetic wave information, greatly affecting their battlefield survival and penetration capabilities. Therefore, there is an urgent need to develop stealth-protective materials that can suppress infrared radiation, reduce acoustic characteristics, and weaken electromagnetic signals. Fibrous three-dimensional porous materials, with their high porosity, excellent structural adjustability, and superior mechanical properties, possess strong potential for development in the field of stealth protection. This article introduced and reviewed the characteristics and development process of fibrous three-dimensional porous materials at both the micrometer and nanometer scales. Then, the process and characteristics of preparing fibrous three-dimensional porous materials through vacuum forming, gel solidification, freeze-casting, and impregnation stacking methods were analyzed and discussed. Meanwhile, their current application status in infrared, acoustic wave, and radar stealth fields was summarized and their existing problems and development trends in these areas from the perspectives of preparation processes and applicability were analyzed. Finally, several prospects for the current challenges faced by fibrous three-dimensional porous materials were proposed as follows: functionally modifying fibers to enhance their applicability through self-cross-linking; establishing theoretical models for the transmission of thermal energy, acoustic waves, and electromagnetic waves within fibrous porous materials; constructing fibrous porous materials resistant to impact, shear, and fracture to meet the needs of practical applications; developing multifunctional stealth fibrous porous materials to confer full-spectrum broadband stealth capability; and exploring the relationship between material size and mechanical properties as a basis for preparing large-scale samples that meet the application’s requirement. This review is very timely and aims to focus researchers’ attention on the importance and research progress of fibrous porous materials in the field of stealth protection, so as to solve the problems and challenges of fibrous porous materials in the field of stealth protection and to promote the further innovation of fibrous porous materials in terms of structure and function.

## 1. Introduction

The continuous progress of detection technology and sensing technology has brought great convenience to human life and industrial production [1]. However, under the urgent situation of information warfare, the survivability of battlefield targets is facing major challenges. Among many detection technologies, infrared detection [2], acoustic detection [3], and radar detection [4] are the most commonly used methods, and they are also the easiest to capture target information. As the most powerful anti-detection means, stealth technology has always been a research hotspot [5,6,7]. Therefore, it is a long-term goal for researchers to prepare good infrared stealth, acoustic stealth, and radar stealth materials.

**Figure 1:**
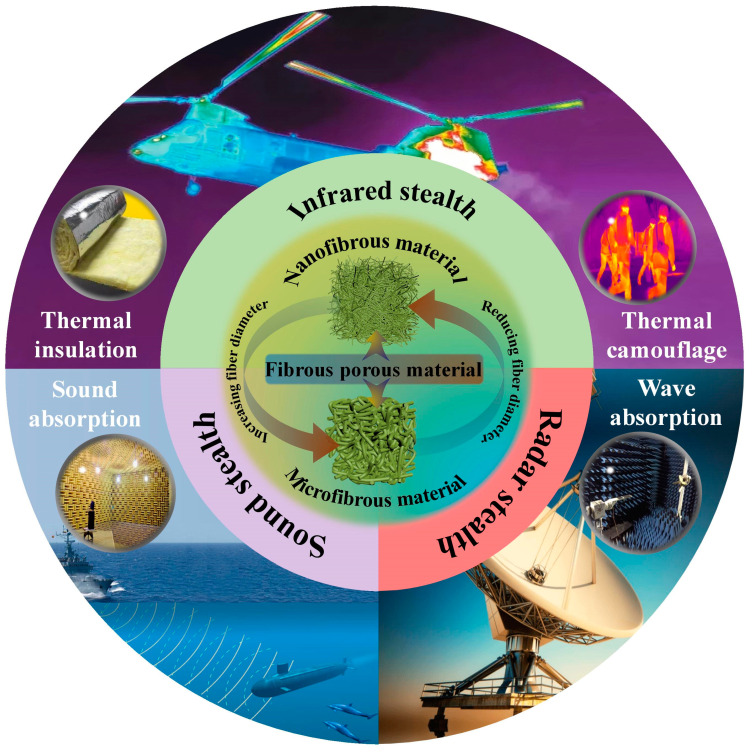
Composition of fibrous three-dimensional porous materials and their application in the field of stealth.

Fibrous three-dimensional porous materials use fibers as monomers, employing a binder or cross-linking to solidify and reinforce fiber overlaps, forming a three-dimensional porous structure similar to a “bird’s nest”. Due to their unique structure and excellent adjustability, they have garnered widespread attention in the field of stealth protection [8,9,10]. The strong connection of fiber overlaps replaces the weak neck-to-neck contact between traditional granular porous materials, improving the mechanical properties of the materials and avoiding the generation of dust particles [11]. Additionally, using one-dimensional fibers instead of zero-dimensional particles as the basic unit reduces the surface energy and prevents structural collapse caused by sintering. Meanwhile, fibrous three-dimensional porous materials also possess lightweight characteristics and considerable flexibility. With excellent structural and functionalized properties, fibrous three-dimensional porous materials have broad application prospects in various fields such as thermal insulation, dynamic thermal camouflage, sound absorption, and electromagnetic wave absorption [12,13,14,15] (Figure 1).

This paper, combining the current research status and hotspots both domestically and internationally, first introduces the classification of fibrous three-dimensional porous materials and discusses their respective characteristics and development histories. Then, based on different molding methods, it enumerates common preparation techniques and discusses the advantages and disadvantages of each method. Finally, it systematically and particularly presents the research progress of fibrous three-dimensional porous materials in the fields of infrared stealth, acoustic stealth, and radar stealth; meanwhile, it summarizes and anticipates their future development trends.

## 2. Material Types

The basic constituent unit of fibrous three-dimensional porous materials is different types of fibers, and varying scales of fibers have a significant impact on the final assembled fibrous three-dimensional porous materials. Therefore, they can be classified into microfibers and nanofibers based on their diameter size. When constructing fibrous three-dimensional porous materials, inorganic ceramic fiber cotton is often used for microfibers, while nanofibers are commonly prepared using methods such as electrospinning [16,17,18], chemical vapor deposition [19,20,21], or derived from biomass [22,23,24]. Due to the relatively large diameter of microfibers, typically ranging from 3 to 100 μm, the pores between the three-dimensional skeletons they form are mostly at the micrometer level. Therefore, they can be further named as microfibrous three-dimensional porous materials. Microfibers below 3 μm in diameter are less commonly studied due to increased preparation difficulty and cost. On the other hand, nanofibers can have diameters below the micrometer level, typically ranging from 10 to 800 nm, and their three-dimensional skeletons contain more nanoscale pores. Hence, they can be further designated as nanofibrous three-dimensional porous materials.

### 2.1. Microfibrous Three-Dimensional Porous Materials

#### 2.1.1. Characteristics

Microfibrous three-dimensional porous materials are typically assembled from ceramic fibers with micrometer-scale diameters. Due to the high strength and stiffness characteristics of microfibers, their assembly into three-dimensional porous materials exhibits limited flexibility, pronounced brittleness, and high strength but poor resilience. Most scholars commonly refer to them as porous ceramic materials [25,26,27,28]. Simultaneously, because most of their skeletons and pores are at the micrometer scale, both the thermal conduction and convection effects are relatively high, resulting in a relatively high thermal conductivity. The extensive literature research indicates that their thermal conductivity at room temperature is generally between 0.03 and 0.2 $W\cdot m^{-1}\cdot K^{-1}$. Additionally, the large external surface area of microfibers allows for the growth of various nanostructures on their surface through a series of methods, further enhancing the complexity of their internal pores.

Due to the high density of microfibers, using a low-density liquid as a dispersant during material formation can lead to fiber sedimentation. Researchers often use substances like starch [27,29] and protein [30] as low-temperature binders and dispersants to prepare microfibrous three-dimensional porous materials. These substances can rapidly solidify the fiber suspension under certain temperature or catalyst conditions. Subsequently, through drying and sintering processes, a uniform fiber assembly with high strength can be obtained.

#### 2.1.2. Development Process

**Figure 2:**
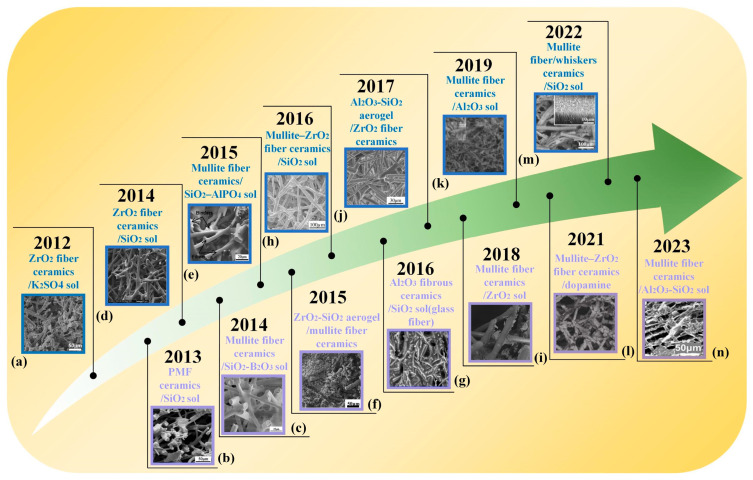
Development of microfibrous three-dimensional porous materials. (**a**) Reproduced with permission [34]; (**b**) reproduced with permission [35]; (**c**) reproduced with permission [37]; (**d**) reproduced with permission [36]; (**e**) reproduced with permission [38]; (**f**) reproduced with permission [39]; (**g**) reproduced with permission [40]; (**h**) reproduced with permission [41]; (**i**) reproduced with permission [42]; (**j**) reproduced with permission [43]; (**k**) reproduced with permission [44]; (**l**) reproduced with permission [45]; (**m**) reproduced with permission [46]; (**n**) reproduced with permission [47].

Microfibrous three-dimensional porous materials, often referred to as rigid insulating tiles, emerged as early as the last century. Their development can be divided into three stages. In the first stage, quartz fibers were used as the skeleton and silica sol served as the high-temperature binder [31]. In the second stage, high-temperature-resistant aluminum borosilicate fibers were added to the quartz fibers [32]. In the third stage, infrared-shielding material alumina fibers were introduced on the previous basis, resulting in a continuous increase in the operating temperature [33]. As illustrated in Figure 2, in recent years, researchers have begun to adopt more mature processes and fibers with better performance to construct microfibrous three-dimensional porous materials [34,35,36,37,38,39,40,41,42,43,44,45,46,47]. It can be seen that the selection of fiber and binder types has always been the focus of research. However, after 2020, basic research on fiber types and binder selection has basically reached saturation. Scholars are committed to changing the structure and performance of the fibers themselves [25,26,48] or preparing functionalized biomimetic microfiber porous materials [27,47], and the research direction tends to be consistent with nanofibrous porous materials.

### 2.2. Nanofibrous Three-Dimensional Porous Materials

#### 2.2.1. Characteristics

The basic component unit of nanofibrous three-dimensional porous materials is generally fibers with diameters at the nanoscale. The small-scale effect of these fibers often gives them flexibility, and thus the three-dimensional porous materials assembled from them also possess flexible and resilient characteristics. However, the low density of nanofibers inevitably leads to a lower mechanical strength compared to microfibrous three-dimensional porous materials. Most scholars commonly refer to them as aerogel materials [49,50,51]. Nanofibrous three-dimensional porous materials contain numerous nanopores, and thermal convection effects are almost nonexistent [52]. The thinner fiber skeleton hinders solid-phase heat transfer. Numerous literature surveys have shown that at room temperature, their thermal conductivity is relatively low, generally ranging from 0.018 to 0.035 $W\cdot m^{-1}\cdot K^{-1}$, which is lower than most microfibrous three-dimensional porous materials.

Nanofibers have lightweight characteristics and unlike high-density microfibers, they are less prone to sedimentation in slurries. However, the nanoscale effect endows them with a higher surface energy, leading to aggregation during dispersion. Dispersants chosen must serve the dual purpose of thickening the slurry and repelling the fibers, making the dispersion process of nanofibers more complex. Currently, most researchers choose to use ionic surfactants (such as Sodium Dodecyl Benzene Sulfonate (SDBS)) or polymeric surfactants (such as polyacrylamide (PAM), polyethylene Oxide (PEO), polyvinyl alcohol (PVA)) [53,54,55,56,57] as dispersing agents for different fiber slurry states. These agents ensure good suspension and dispersion of the fibers through charge repulsion and thickening effects, guaranteeing the uniformity and structural stability of the final sample.

#### 2.2.2. Development Process

**Figure 3:**
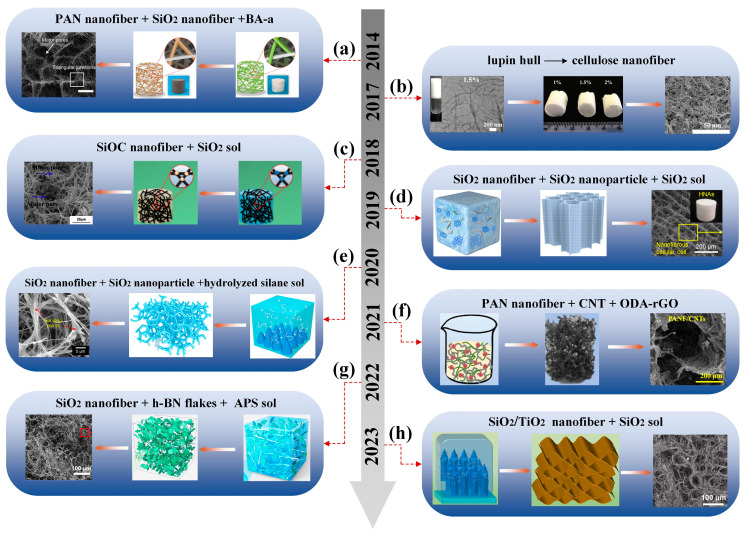
Development of nanofibrous three-dimensional porous materials. (**a**) Reproduced with permission [58]; (**b**) reproduced with permission [59]; (**c**) reproduced with permission [60]; (**d**) reproduced with permission [54]; (**e**) reproduced with permission [61]; (**f**) reproduced with permission [50]; (**g**) reproduced with permission [62]; (**h**) reproduced with permission [63].

Compared with microfibers, nanofibers have significantly fewer defects, and the nano-size effect gives the material functionalized characteristics, making it more adaptable to the complex and changing environment. Over the past 10 years, nanofibrous three-dimensional porous materials have made significant progress [50,54,58,59,60,61,62,63], as shown in Figure 3. It can be seen that their development has gradually shifted from the assembly of single nanofibers into a three-dimensional framework to the optimization of nanofiber structural components, composite assembly with other two-dimensional sheet materials and three-dimensional aerogel materials, with more attention paid to other functionalized characteristics (such as acoustic properties, wave absorption properties, sensing properties, etc.).

## 3. Construction Method of Fibrous Three-Dimensional Porous Materials

There are many methods for forming fibrous porous materials, including fluffy spongy materials directly obtained by the electrostatic spinning method and with the body receiver [64,65,66,67], and stacked nanowire materials directly obtained by the high-temperature vapor deposition method on the crucible [19,68], and the fibrous three-dimensional porous materials directly obtained by these methods are only for the stacked assembly of fibers and random deposition, which lacks the cross-linking and stable structure of the inter-fibers [58]. The main discussion in this paper is to assemble existing fiber monomers into fibrous three-dimensional porous materials with a certain strength and wider applications by the physical and chemical bonding of binders, or by enhancing their fiber lapping points through stronger intermolecular forces or the cross-linking of functional groups between fibers. Currently, the most common preparation methods include vacuum forming, gel solidification, freeze-casting, and impregnation stacking.

### 3.1. Vacuum-Forming Method

**Figure 4:**
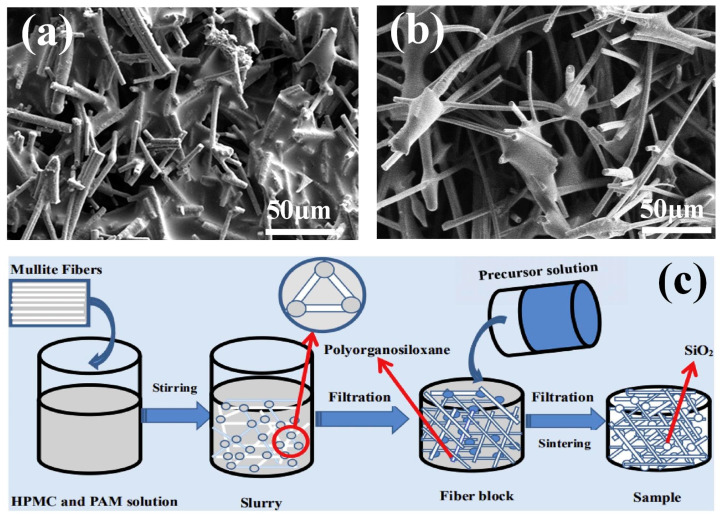
Preparation of fibrous porous materials by vacuum forming method. (**a**,**b**) SEM image of fibrous ceramics with silica sol and glass fibers as the high-temperature binder [40]; (**c**) flow chart for the preparation of mullite fiber porous ceramics by vacuum-forming method [70].

The vacuum-forming method was initially used by researchers to prepare three-dimensional porous fiber materials due to its simple operation and rapid molding. The main process involves draining excess slurry through a filter using a vacuum-filtration device. The remaining slurry encounters high resistance at the fiber overlap, forming stable nodes, and eventually forms a porous structure assembled by fibers under pressure. Inspired by the structure of bird’s nests in nature, Dong et al. [69] synthesized mullite fibrous porous ceramics using polycrystalline mullite fibers as the matrix and silicon boron sol as a high-temperature binder, employing the vacuum-molding method. However, during high-temperature sintering, the silicon boron glass phase can severely melt, affecting the structural stability. The research team switched to a SiO_2_-AlPO_4_ binder with better high-temperature stability [38], providing better high-temperature stability to the samples. In the vacuum-filtration process, the type of binder affects the resistance when passing through the filter, thereby influencing the final material structure. Zang et al. [40] used alumina fibers as the basic unit and prepared two types of ceramic materials using two high-temperature binders: silica sol and glass fibers. Although materials with silica sol as the high-temperature binder exhibited a higher compressive strength, they could fill the pores between alumina fibers during vacuum filtration, reducing the porosity. Samples with glass fibers as the high-temperature binder had a higher porosity and compressive resilience (Figure 4a,b). To address the thermal migration of silica sol during heating and sintering, Zhu et al. [70] introduced polysiloxane as a high-temperature binder, which decomposes in situ into SiO_2_ during the sintering process, maintaining the stability of fiber nodes during drying (Figure 4c). To further enhance the compressive strength of fibrous porous ceramics, Zhang et al. [71] adopted a vacuum extrusion molding method, selecting B_4_C and SiC as inorganic binders. This approach reduced the sintering temperature while providing the compounds with a higher melting point, resulting in the preparation of high-strength (8.16 MPa) mullite fiber ceramics. This provides insights into the preparation of high-strength fibrous porous ceramics.

### 3.2. Gel Solidification Method

The gel solidification method causes the slurry to set within a short period of time through a curing agent, avoiding the natural drawback of fiber sedimentation due to its own gravity. This ensures that the fibers are “embedded and assembled” in the green body before they settle. The main techniques include gel casting, starch in situ solidification, and protein gel solidification.

**Figure 5:**
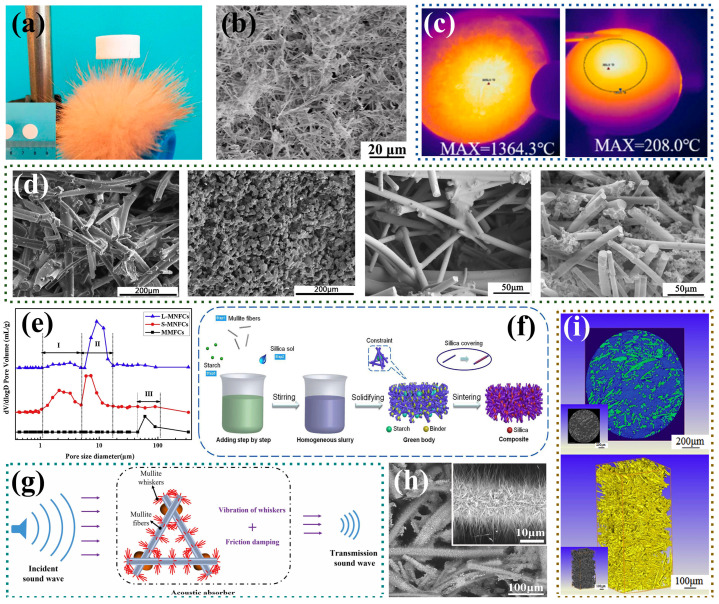
Preparation of fibrous porous materials by gel solidification method. (**a**) Lightweight mullite-based nanofibrous aerogel [72]; (**b**) microstructure of aluminum borate nanofibrous porous ceramics [73]; (**c**) Dy_0.2_Y_0.2_Ho_0.2_Er_0.2_Yb_0.2_)_2_Zr_2_O_7_ high-entropy nanofibrous porous ceramics with excellent high-temperature thermal insulating properties [76]; (**d**) microstructures of mullite fibrous ceramics with different fiber lengths and solid-phase contents [78]; (**e**) pore size distribution of mullite fibrous porous ceramics with different lengths and diameters [79]; (**f**) flow chart of the preparation of fibrous porous ceramics by starch in situ solidification method [29]; (**g**,**h**) schematic diagram of the acoustic absorption mechanism and SEM image of mullite fibrous porous ceramics with in situ growth of mullite whiskers [46]; (**i**) XRT image of mullite fibrous porous ceramics [80].

Common gel-forming agents for gel casting are agarose and tert-butyl alcohol (TBA). Liu et al. [72] prepared mullite-based nanofibrous aerogels using electrospinning, agarose gel casting combined with the freeze-drying method, showing a multilevel porous structure with a minor pore size of 0.5–10 μm and a major pore size of 5–100 μm. Their hierarchical pore structure exhibited an ultra-low density and room temperature thermal conductivity (Figure 5a,b). Subsequently, the research team switched to the atmospheric pressure drying process to prepare aluminum borate nanofibrous aerogels with a high compressive strength and low thermal conductivity [73]. As the sintering temperature was increased from 1000 to 1400 °C, the large pores (12 μm) disappeared due to the volatilization of the polymer, and the small pore size (1.3 μm) was preserved almost unchanged. High-entropy ceramics have been developed rapidly in recent years by virtue of their excellent thermal and mechanical properties [74,75]. Shao et al. [76] introduced this concept into fibrous porous ceramics, preparing a high-entropy (Dy_0.2_Y_0.2_Ho_0.2_Er_0.2_Yb_0.2_)_2_Zr_2_O_7_ (DYHEY) nanofibrous porous ceramic for extreme thermal protection using agarose gel casting combined with the freeze-drying method (Figure 5c). Researchers found that the binder, fiber aspect ratio, and solid content affect the pore structure and performance of the fibrous porous ceramics prepared by the TBA-based gel-casting method. Xu et al. [77] discovered that SiO_2_-B_2_O_3_ had a higher compressive strength, while Si particles had a better thermal stability when preparing mullite fiber ceramics with different binders. As shown in Figure 5d, Zhang et al. [78] discussed the influence of solid content on the structure and performance of fiber ceramics. When the solid content was 10%, the thermal conductivity of the fibrous porous ceramic was as low as 0.08 $W\cdot m^{-1}\cdot K^{-1}$. Guo et al. [79] replaced mullite micrometer fibers with mullite nanofibers, increasing the fiber aspect ratio, significantly reducing the average pore size, which decreased from 60 to 6 μm, and improving the thermal insulation performance (Figure 5e).

Methods for starch in situ solidification and protein gel solidification molding are also commonly used to prepare fibrous porous materials. Starch and protein are also natural pore-forming agents, which have a certain regulatory effect on the microstructure of fibrous porous materials. Liu et al. [29] used the constraint effect of starch on fiber orientation to form a better three-dimensional lattice structure of fibers, and investigated the effect of the starch content on the pore structure and thermal insulation properties of the material. The results showed that with the increase in starch content, the pore size increased, and the pore size distribution became narrower. When the content was higher than 50 vol%, the starch would agglomerate, and the range of pore size distribution would become coarsened. In addition, the pore size distribution of the samples with 30 vol% of starch content was the most uniform, with the average pore size of 10 μm and the thermal conductivity as low as 0.035 $W\cdot m^{-1}\cdot K^{-1}$ (Figure 5f). Yang et al. [46] prepared mullite fibrous porous ceramics with in situ grown mullite whiskers using protein gel solidification combined with vacuum impregnation. The median pore size of the sample was reduced from 210.9 to 12.8 μm compared to the sample without whisker growth. Due to the resonance effect of the whiskers, the material’s low-frequency sound absorption performance was significantly improved (Figure 5g,h). Yuan et al. [80] selected ρ-Al_2_O_3_ powder, which can serve as both a gel curing agent and a high-temperature binder. They obtained mullite fibrous porous ceramics through the gel solidification method (Figure 5i). This multifunctional gelling agent reduces raw material usage and simplifies the preparation process, potentially becoming a major trend in the future development of gel solidification methods.

### 3.3. Freeze-Casting Method

**Figure 6:**
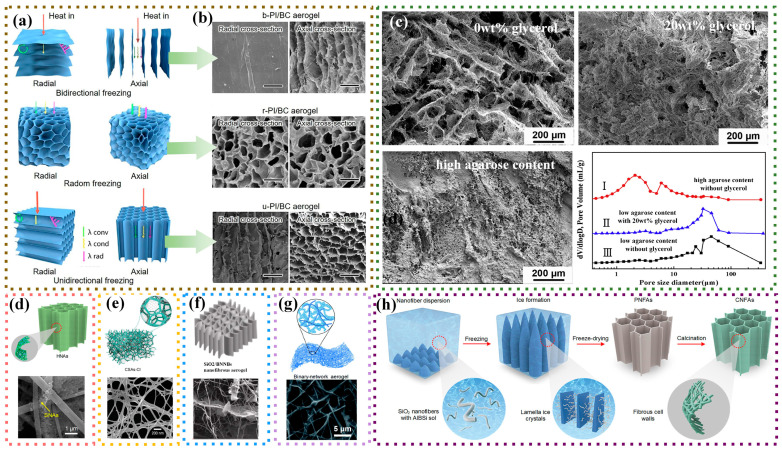
Preparation of fibrous porous materials by freeze-casting method. (**a**,**b**) Schematic structure and SEM images of ice crystals with different freezing methods [82]; (**c**) effect of different ice crystal growth regulators on the structure of fibrous porous materials [89]; (**d**) fibrous porous materials with added aerogel powder [54]; (**e**) fibrous porous materials with added bacterial cellulose [90]; (**f**) fibrous porous materials with added BN nanoribbons (BNNBs) [91]; (**g**) fibrous porous materials with added aerogel [92]; (**h**) flow chart of preparing fibrous porous materials by the freezing and casting method [53].

As a near-net-shape preparation method for biomimetic porous materials, the freeze-casting method, also known as the ice-templating method, involves a process of “dispersion-molding-freeze drying” to produce processable lightweight materials [81]. The general process of the freeze-casting method for fibrous porous materials includes fiber dispersion, dispersion solution freeze-molding, and freeze-drying. Among these steps, the freeze-molding of the dispersion solution has the most significant impact on the pore structure. Zhang et al. [82] compared the effects of three different freeze methods: directional [83,84], bidirectional [85,86], and random freezing [87,88] on the microstructure and properties of polyimide (PI)/bacterial cellulose (BC) aerogels (Figure 6a,b). The samples prepared by directional and random freezing have a pore size of 10–20 μm, and the average pore size of the sample prepared by bidirectional freezing is 10 μm, and the differences in the pore structure determine the variations in their thermal insulation performance and application scenarios. Xian et al. [89] investigated the effects of glycerol and agarose on the microstructure of mullite nanofibrous aerogels; the results showed that the samples without added glycerol showed a multistage pore distribution, and the addition of glycerol affected the growth of ice crystals, which resulted in a decrease in the macropores’ size up to 30 μm. Meanwhile, the formation of the gel network of agarose greatly suppressed the growth of ice crystals, which led to the disappearance of macropores, and the pore size was mainly distributed in the range of 2–6 μm. Figure 6 demonstrated the effect of different glycerol and agarose additions on the microscopic morphology and pore size of the samples (Figure 6c).

The single-cell wall structure of fibrous porous materials prepared by the freeze-casting method limits its application scope. As shown in Figure 6d–g, to broaden its applications, researchers have begun to experiment with adding zero-dimensional [54], one-dimensional [90], two-dimensional [91], and three-dimensional materials [92] during the freeze-casting process. This creates complex multi-dimensional composite fibrous porous materials, enhancing the roughness and complexity of the fiber wall surface. This makes the propagation of thermal and acoustic energy more tortuous within the pores, further expanding its range of applications.

To meet practical demands, sol components have also undergone development from single, binary, to multi-component sols. Wang et al. [93] compared the mechanical properties of three types of fibrous aerogels using single hydrolyzed silane sol as a binder. They found that the methyltrimethoxysilane (MTMS) network, with its advantages in cross-linking degree and flexibility, along with a three-level deformation mechanism, endowed the fibrous aerogel with optimal mechanical properties. Based on this, the research team continued to use binary silane sol to construct a “rigid-flexible synergistic” cross-linked network, improving the overall mechanical properties [61]. To further build a stable fiber cross-linked structure, Yang et al. [53] adopted silicoaluminoborate (AlBSi) sol, widely used in the synthesis of high-temperature ceramic matrices (Figure 6h). This forms a stable bonded structure under high-temperature calcination, resulting in a fibrous aerogel with temperature-invariant super-elasticity.

### 3.4. Impregnation Stacking Method

Inspired by the spring-like lamellar cell structure of woody sponges [94,95], researchers have constructed layered fibrous three-dimensional porous materials by stacking fibrous membranes layer by layer, followed by sol impregnation and freeze-drying. This impregnation stacking method, with its large contact area, endows the fibrous porous material with high strength and super elasticity. Zhang et al. [96] combined flexible ZrO_2_-Al_2_O_3_ nanofibers with aluminum dihydrogen phosphate (Al(H_2_PO_4_)_3_) sol, using the impregnation stacking method to prepare nanofibrous aerogels with an anisotropic lamellar structure and a room temperature thermal conductivity of 0.0322 $W\cdot m^{-1}\cdot K^{-1}$. However, the tight packing between fiber membrane layers results in limited deformation space and energy absorption capacity. The same research team then utilized an ultrasonic-assisted impregnation stacking method and replaced it with more flexible ZrO_2_-SiO_2_ nanofibers to prepare nanofibrous aerogels with a rich arched cell structure [97]. After 1000 cycles, there was no plastic deformation, and the room temperature thermal conductivity was reduced to 0.0268 $W\cdot m^{-1}\cdot K^{-1}$, which is comparable to that of air. The single-fiber membrane stacked structure is still dominated by micrometer pores with few nanometer pores. Therefore, Zhang and his team [98] added SiO_2_ nanoparticles during the impregnation stacking process, filling the micrometer pores and significantly reducing the thermal conductivity (0.024 $W\cdot m^{-1}\cdot K^{-1}$).

**Figure 7:**
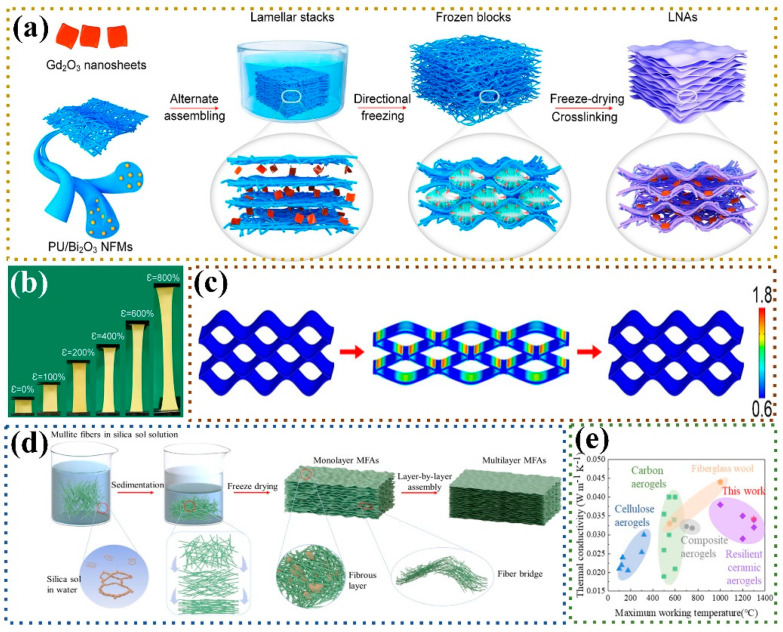
Preparation of fibrous porous materials by impregnation stacking method. (**a**–**c**) Flow chart of the preparation and tensile process analysis of Gd_2_O_3_ nanosheets composite PU/Bi_2_O_3_ nanofibrous aerogel [99]; (**d**) preparation of mullite nanofibrous aerogel by tunable fiber deposition and impregnated stacking nanofibrous aerogels by adjustable fiber deposition and impregnation stacking [100]; (**e**) excellent thermal insulation properties of mullite nanofibrous aerogels [100].

The fibrous porous materials prepared by the impregnation stacking method have a strong compression performance, but their tensile properties are often overlooked. Inspired by the layered brick/mortar structure of nacre layers, Ding et al. [99] combined polyurethane (PU)/bismuth trioxide (Bi_2_O_3_) nanofibers and gadolinium oxide (Gd_2_O_3_) nanosheets to prepare nanofibrous aerogels with a micro-arched brick/mortar structure (Figure 7a). The straightening deformation ability of the ordered micro-arched structure and the elastic stretching ability of nanofibers endow the nanofibrous aerogel with 800% reversible elongation (Figure 7b,c). Li et al. [100] proposed a method combining fiber deposition and impregnation stacking to prepare layered mullite fibrous aerogels with adjustable fiber orientation (Figure 7d). Fibers arranged horizontally ensure good flexibility and elasticity of the material, while fibers bridged at an angle to the horizontal direction endow the material with good mechanical strength. The ceramic composition also gives the material excellent thermal insulation properties (Figure 7e). This method increases the adjustability and scalability of fiber orientation, and the prepared quasi-ordered structure maintains good flexibility while maintaining a high mechanical strength, providing a unique new idea for subsequent research.

## 4. Application of Fibrous Three-Dimensional Porous Materials in Stealth Field

After years of research, fibrous three-dimensional porous materials have gradually developed towards hierarchical, nanoscale, and composite directions. Their unique micro-nano pore structure, complex fiber wall structure, good elastic recovery ability, and machinability/scalability endow fibrous porous materials with various inherent advantages, greatly broadening their application in stealth protection. They have been widely reported in infrared stealth [15,48,101], acoustic stealth [102,103], and radar stealth [104,105].

### 4.1. Infrared Stealth

All objects in nature emit energy [106], and passive infrared detection is based on this principle to detect the location of the target, thereby achieving precise guidance. Therefore, the infrared stealth performance of weapons and equipment is particularly important. Weapons and equipment with excellent infrared stealth performance can blend themselves into the surrounding environment very well, thus confusing the enemy and achieving the goal of winning in one strike. According to Stefan–Boltzmann’s law [107], the radiation power (*E*) of an object in all possible directions and wavelength ranges is

(1) $$E=\varepsilon \sigma T^{4}$$

where ε is the emissivity of the object, σ is the Stefan–Boltzmann constant, and T is the thermodynamic temperature (in Kelvin). To change the radiation energy of an object, it is necessary to adjust its own temperature and the emissivity of the object. Infrared stealth is mainly divided into static infrared stealth and dynamic infrared stealth in terms of application.

#### 4.1.1. Static Infrared Stealth

Traditional infrared stealth materials mainly target static stealth materials. They primarily conceal themselves by lowering the target temperature to reduce the thermal radiation energy. Generally, they can achieve excellent camouflaging effects under stable ambient conditions, emphasizing the material’s insulation and heat-preservation capabilities. Fibrous porous materials have unique advantages in reducing heat loss. Their heat transfer process includes thermal conduction, thermal convection, and thermal radiation. The effective thermal conductivity can be represented using the formula

(2) $$\lambda_{\mathrm{total}}=\lambda_{\mathrm{cond}}^{g}+\lambda_{\mathrm{cond}}^{s}+\lambda_{\mathrm{conv}}+\lambda_{\mathrm{rad}}$$

where $\lambda_{\mathrm{total}}$, $\lambda_{\mathrm{cond}}^{g}$, $\lambda_{\mathrm{cond}}^{s}$, $\lambda_{\mathrm{conv}}$, and $\lambda_{\mathrm{rad}}$ represent the overall thermal conductivity, gas-phase conduction, solid-phase conduction, thermal convection, and thermal radiation, respectively. Gas-phase conduction is mainly affected by the size of the fiber pores, generally decreasing as the pore size decreases. Solid-phase conduction is primarily influenced by the fiber’s own properties, with both the diameter size and internal structure affecting heat transfer. Thermal convection is generally more pronounced in porous materials with pores larger than 1 mm, and can be neglected in smaller pores, especially when the pore size is smaller than the mean free path of air molecules (70 nm), at which point thermal convection ceases to exist. Thermal radiation is less affected by the temperature at room temperature, but at high temperatures, it becomes more significantly influenced by the temperature, and its proportion increases.

**Figure 8:**
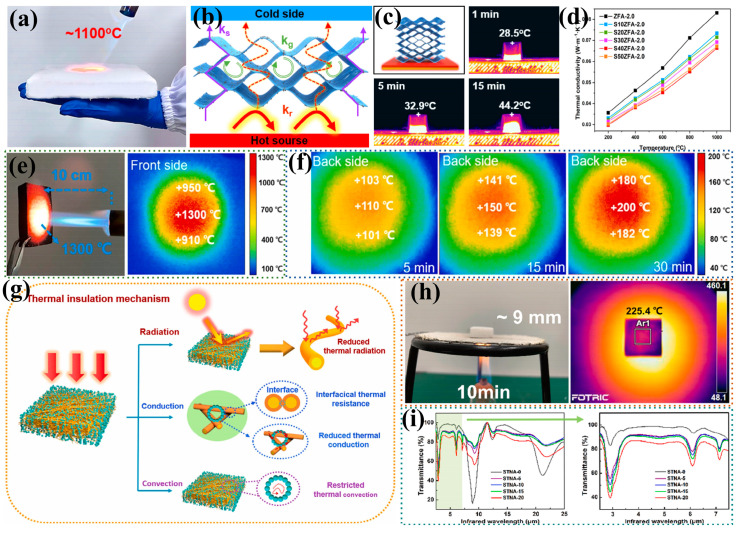
Application of fibrous porous materials in the field of heat insulation. (**a**) SZFA undamaged under flame at 1100 °C [111]; (**b**) thermal insulation mechanism of SZFA [111]; (**c**) heating experiment of SZFA on a flat plate at 300 °C [111]; (**d**) high-temperature thermal conductivity of SZFA with different SiC contents [111]; (**e**,**f**) temperature change in NF/SiBCN hot and cold surfaces under butane flame at 1300 °C [112]; (**g**) thermal insulation mechanism of NF/SiBCN [112]; (**h**) excellent thermal insulation properties of STNA under alcohol lamp burning [63]; (**i**) infrared transmittance of STNA with different TiO_2_ contents [63].

Weaponry and equipment are usually self-heating during operation and generally operate at temperatures well above room temperature. Therefore, high-temperature insulation performance requires special attention in infrared stealth. Researchers often reduce the infrared transmittance of materials and enhance their high-temperature insulation properties by adding infrared shading agents such as SiC [108,109] and TiO_2_ [64,110]. Feng et al. [111] prepared SiC particle composite zirconia fiber aerogels (SZFA) using the directional freeze-casting method. The thermal conductivity at 1000 °C is only 0.0663 $W\cdot m^{-1}\cdot K^{-1}$, which is much lower than that of pure zirconia fiber aerogels, and it remains undamaged under flame heating at 1100 °C (Figure 8a). The reason is that the addition of an appropriate amount of SiC particles increases the material’s density and specific extinction coefficient, ensuring that the density of the fiber skeleton is not too low to be sintered and fail, thereby significantly reducing the radiative thermal conductivity (Figure 8b). It exhibits excellent insulation performance over a wide temperature range (Figure 8c,d) and is expected to be applied in the field of infrared stealth. Zhong et al. [112] added the opaque agent TiO_2_ component to the spinning solution and prepared nanoparticle fiber/SiBCN composite aerogels (NF/SiBCN) using electrostatic spinning and impregnation processes. Since the sample contains SiBCN aerogel components and has a rich mesoporous structure, the pore sizes of all samples are distributed in the range of 4–50 nm. At the same time, due to the support of the fiber skeleton, the specific surface area of the sample did not change much with the increase in the pyrolysis temperature, and the specific surface area was still as high as 700 $m^{2}\cdot g^{-1}$ at 1600 °C. By weakening heat conduction, heat convection and thermal radiation processes, NF/SiBCN is ensured to have excellent high-temperature insulation properties (Figure 8e–g). To eliminate the drawbacks of particle release during the compositing of aerogels with fibers, Ding et al. [63] embedded a TiO_2_ aerogel during the preparation of SiO_2_ nanofibers by electrostatic spinning, resulting in a SiO_2_/TiO_2_ nanofibrous aerogel (STNA). After treatment at 600 °C, the TiO_2_ aerogel still maintained a high specific surface area of 557 $m^{2}\cdot g^{-1}$, and the pore size was concentrated in the range of 3–55 nm, with an average pore size of 10 nm, which endowed the sample with good mesoporosity and greatly reduced the gas-phase heat conduction and thermal convection of the material. Meanwhile, the infrared shading effect of TiO_2_ improved the high-temperature thermal insulation performance of the samples, and the samples exhibited excellent infrared-radiation-suppression performance (Figure 8h,i), which effectively improved the high-temperature infrared stealth performance of the materials.

The interior of fibrous porous materials is mostly composed of overlapping pores created by the fibers, making it difficult to achieve a mesoporous structure. Therefore, their thermal conductivity is rarely below 0.020 $W\cdot m^{-1}\cdot K^{-1}$, which affects the further improvement in their insulation and heat-preservation properties. Filling and segmenting the internal pores of fibrous porous materials with finer BC [63,113,114,115], cellulose nanofibers (CNF) [24,116] to reduce pore size is a trend for future development. When the fiber diameter cannot be further reduced, another approach is to construct an aerogel structure or prepare hollow fibers inside, making the heat transfer path more complex and gradually improving the material’s insulation performance. However, how to balance the mechanical properties and insulation performance of this fibrous porous material is an urgent problem that needs to be solved.

#### 4.1.2. Dynamic Infrared Stealth

**Figure 9:**
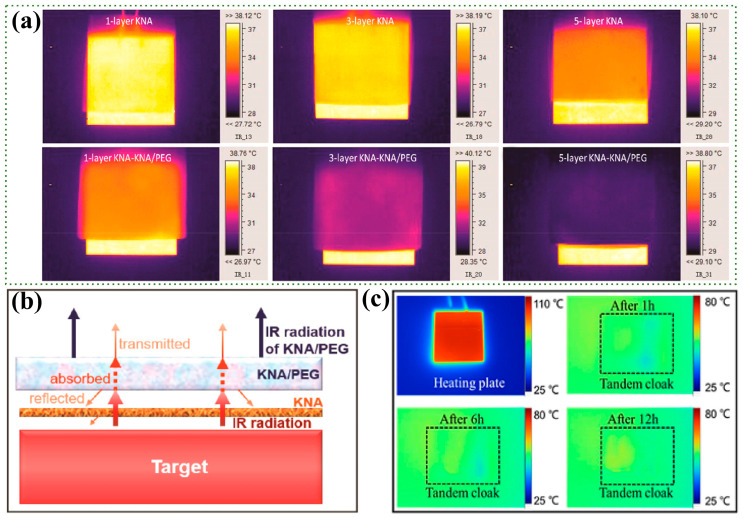
Application of fibrous porous materials in the field of dynamic thermal camouflage. (**a**) Synergistic infrared stealth performance of KNA film and KNA/PEG film [12]; (**b**) schematic diagram of the infrared stealth mechanism of KNA and KNA/PEG against a thermal target [12]; (**c**) original images of the heated plate covering the tandem cloak and infrared images after 1 h, 6 h, and 12 h of sunlight irradiation [119].

The battlefield environment is constantly changing, potentially shifting from daylight to darkness. As a result, the radiant energy of the environment is also dynamically changing. Merely relying on thermal insulation for the target may not ensure consistency with the environment’s radiant energy. Changes in the surrounding environment may cause the target to be detected by infrared sensors and precisely guided. Therefore, dynamic infrared stealth (dynamic thermal camouflage) is more aligned with actual battlefield conditions and meets the demands of modern warfare. Phase-change materials with latent heat storage capabilities can achieve the effect of dynamic thermal camouflage [117,118]. Fibrous porous materials, with their excellent thermal insulation properties, micro-nano porous structure, shape stability, and encapsulation, are excellent materials for addressing this issue. Lyu et al. [12] proposed the idea of combining Kevlar aramid fiber aerogels (KNAs) with the phase-change material polyethylene glycol (PEG) for infrared stealth, and successfully prepared related composites. Whether through supercritical drying or freeze-drying, the KNAs maintained a high specific surface area (272.52–365.99 $m^{2}\cdot g^{-1}$) and possessed a rich mesoporous structure (11.69–15.97 nm), which endowed the material with excellent thermal insulation properties and provided enough space for the encapsulation of the phase-change material at the same time. They simulated the dynamic infrared stealth characteristics of self-heating targets and found that adding a KNA insulation layer between the KNA/PEG film and the target can further reduce the difference in thermal radiation between the target and the background (Figure 9a,b).

Subsequently, the team designed a three-layer series cape based on Kevlar nanofibrous aerogels [119]. The top layer mainly exhibits low infrared emissivity (0.12) and high solar reflectivity (0.87). The middle layer, achieved by infiltrating paraffin into the KNA film, is used to store the thermal energy escaping from the top or bottom layers. The bottom layer boasts low infrared transmittance, high thermal reflectivity (0.94), and high thermal resistance (10.42 $K\cdot W^{-1}$). With a good mesoporous structure (average pore size of 20–30 μm) and a large specific surface area (256 $m^{2}\cdot g^{-1}$), this three-layer series cape enables efficient dynamic infrared stealth under heating and sunlight exposure (Figure 9c). Additionally, some researchers have developed hollow fibers [8,120,121] and infiltrated phase-change materials into their interiors and gaps, significantly increasing their loading capacity. Phase-change fibrous porous materials represent a new approach to enhancing dynamic thermal camouflage performance.

Currently, most research on infrared stealth materials focuses on static infrared stealth, which meets the requirements of infrared stealth in general situations. However, as application scenarios become more complex, dynamic thermal camouflage represents the future direction of development. The utilization of advanced functions of phase-change materials is still in its infancy, and their composite application with fibrous porous materials for infrared stealth has just begun, with limited research available. Therefore, researchers need to deeply understand the inherent characteristics of phase-change materials and pay more attention to dynamic infrared stealth performance in complex backgrounds.

### 4.2. Acoustic Stealth

Excellent acoustic stealth materials can enhance combat effectiveness while concealing position and improving battlefield survivability. Therefore, it is crucial to develop acoustic stealth materials with sound absorption properties.

**Figure 10:**
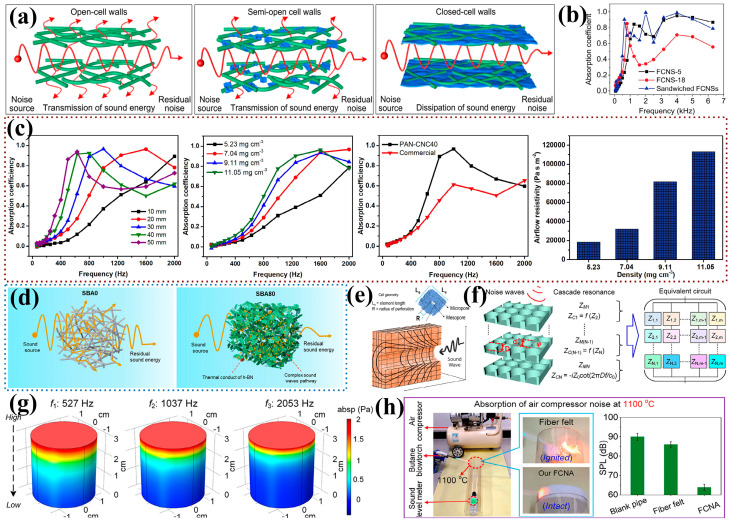
Application of fibrous porous materials in the field of sound absorption. (**a**) Schematic sound propagation mechanism of three pore–wall structures [122]; (**b**) acoustic performance of fiber aerogels with three different densities and structures [122]; (**c**) effects of different thicknesses and densities on the acoustic performance of PAN–CNC aerogels [123]; (**d**) schematic sound absorption mechanism of SBA before and after the introduction of h-BN [62]; (**e**) schematic of sound wave propagation in micro–perforated porous media [124]; (**f**–**h**) geometry, transmission impedance calculations, simulated absolute sound pressure distribution, and practical noise testing of cascade resonance FCNAs [127].

Refining the fiber diameter can increase the specific surface area of the material, thereby increasing the frictional contact area between sound waves and the wall surface and promoting the dissipation of sound energy. Current research tends to focus on sub-micron fibers and nanofibers. Introducing two-dimensional nanosheets with a large aspect ratio into fibrous porous materials can block sound propagation, making it a good approach for preparing sound-absorbing materials. As shown in Figure 10a, Zong et al. [122] achieved the robust preparation of flexible ceramic nanofibrous aerogels with a hierarchical entangled graphene network through a combination of directional freeze-drying and ascorbic acid reduction methods. As the proportion of nanosheets increases, the wall gradually transforms into a closed-cell wall, and the airflow resistance increases accordingly. Additionally, nanofibrous aerogel composites with different density sandwich structures were prepared, achieving a noise reduction coefficient (NRC) of 0.56 and exhibiting a good sound absorption performance across a wide frequency band (Figure 10b). Inspired by the concepts of labyrinth structures and divided spaces, Cao et al. [123] designed a nanofibrous aerogel with a labyrinth structure. Randomly oriented cellulose nanocrystal lamellae (CNC) divide the internal space of the polyacrylonitrile (PAN) nanofibrous aerogel, internally presenting a multistage pore labyrinth structure with the coexistence of both large (50–100 μm) and small (10 μm) pore sizes, which increased the tortuosity of the sound wave transmission path. Increasing the material density and thickness enhances the low-frequency sound absorption capability, and the nanofibrous aerogel exhibits excellent performance (with an NRC of up to 0.58), providing a new concept for the design of efficient fibrous sound-absorbing materials (Figure 10c). The research team further developed a fire-resistant layered structure elastic ceramic nanofibrous aerogel using flexible and non-flammable SiO_2_ nanofibers and boron nitride nanosheets (h-BN) [62]. As shown in Figure 10d, the introduction of h-BN prevents sound waves from directly transmitting through the fiber pores, increasing sound wave dissipation internally. This material not only has good acoustic stealth performance but can also block fire sources, making it suitable for sound absorption and noise reduction in harsh environments.

In addition to dividing the space to increase the dissipation path of sound waves, micro-perforated plates’ resonance structure sound-absorbing materials also exhibit an efficient sound absorption performance. Puguan et al. [124] drew inspiration from the sound absorption characteristics of micro-perforated plates and proposed a micro-perforated PET nanofibrous aerogel. Compared to unperforated aerogels, the sound absorption coefficient at 2000 Hz increased from 0.83 to 0.9. When the diameter of the perforation was increased from 1 to 3 mm, the pressure diffusion effect was more obvious, and the acoustic performance was improved (Figure 10e). Constructing a resonant cavity structure is another way to improve the low-frequency sound absorption performance [125,126]. Zong et al. [127] developed a cascaded resonant cavity structure flexible ceramic nanofibrous aerogel (FCNAs) using bubble-assisted freeze-casting technology (Figure 10f,g). The pore size of the FCNAs increased with the increase in the bubble size, which will decrease the porosity and improve the inhomogeneity. By controlling the stirring rate to 6 $\mathrm{kr}\cdot \min^{-1}$, the most structurally homogeneous samples were obtained, which showed good practical noise absorption at both room temperature (25 °C) and high temperature (1100 °C) (Figure 10h). Yang et al. [46] grew mullite whiskers in situ on the surface of mullite fibers, enhancing the material’s low-frequency sound absorption capability due to the resonance effect of the whiskers.

Although fibrous porous materials have achieved some success in the field of acoustic stealth, there are still many challenges. Low-frequency noise attenuates more slowly, travels further, and is more easily detected by sonar. Improving the sound absorption performance in the ultra-low-frequency range below 200 Hz remains a difficult challenge, and it is difficult to achieve this solely through material structure adjustments. There is a tendency to construct resonant pore structures, so further exploration is needed into the resonance sound absorption mechanism of fibrous porous materials. Increasing the fiber surface roughness can effectively improve the sound absorption performance. However, this method is currently limited to micron fibers, and in situ growth technology on nanofiber surfaces is a bottleneck that needs to be broken through. Additionally, aerogel fibers [128,129] can also enhance the complexity of the fiber surface and interior. However, their mechanical properties must meet practical application conditions before they can be used.

### 4.3. Radar Stealth

With the development of radar technology and microwave weapons, the radar stealth performance and electromagnetic wave interference resistance of weapon equipment have attracted particular attention. Preventing precise positioning by radar detection and precise strikes by microwave weapons is essential to ensure combat effectiveness. Researchers aim to develop radar stealth materials with characteristics such as thin thickness, lightweight, broadband width, and strong absorption [130]. The three-dimensional network structure of fibrous porous materials can provide more complex paths for electromagnetic wave dissipation, generating more reflection and scattering to enhance electromagnetic wave absorption [4].

**Figure 11:**
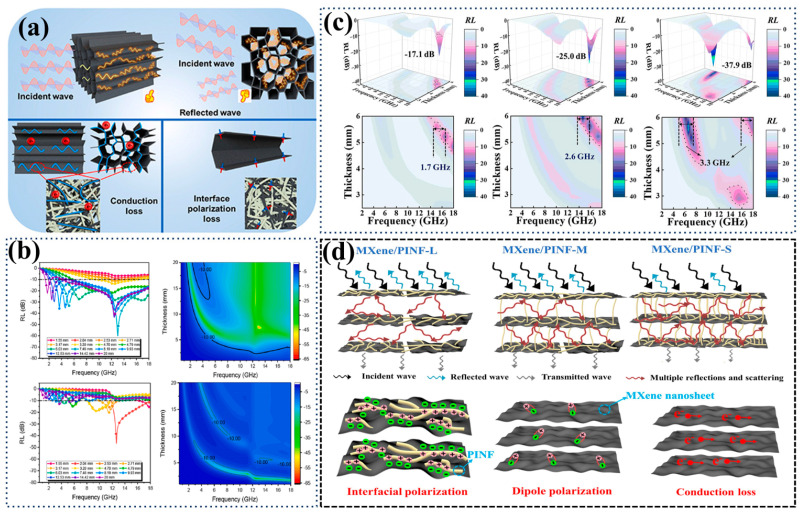
Application of fibrous porous materials in the field of electromagnetic wave absorption. (**a**) Wave absorption mechanism for UCCA [132]; (**b**) axial and radial absorbing properties for UCCA–25 [132]; (**c**,**d**) wave absorption properties and mechanisms of three types of MXene/PINF aerogels [134].

Carbon-based composite fibrous porous materials have good environmental adaptability and a wide range of raw material sources, making them widely used in absorbing electromagnetic waves [13]. Liu et al. [131] used abundant chitin as a raw material to prepare a chitin-derived carbon nanofibrous aerogel, successfully achieving strong microwave absorption within a single carbon material. With a minimum reflection loss (RL_min_) of −92.8 dB at 11.6 GHz, it serves as an excellent radar stealth material. Huang et al. [132] achieved the preparation of a mixed double-network aerogel (UCCA) of CNF and carbon nanotubes (CNTs) through unidirectional circular freezing and thawing. Figure 11a illustrates the dissipation mechanism of electromagnetic waves in the UCCA. A low CNT content contributes little to microwave absorption, while an excessively high CNT content leads to the microwave being reflected on the surface rather than entering and being absorbed within the UCCA. Consequently, with a specific surface area of 32.7 $m^{2}\cdot g^{-1}$ and an average pore size of 108 μm, UCCA–25 exhibited the optimal wave-absorbing performance. The relationship between the reflection loss in different directions, thickness, and frequency was investigated, as shown in Figure 11b, and the composite aerogel exhibited an RL_min_ of −60.22 dB at 13.01 GHz and an effective absorption bandwidth (EAB) of 15.837 GHz at 12.53 mm, covering almost all bands of S, C, X, and Ku, which makes it an ideal choice for full-bandwidth radar stealth materials.

In addition to carbon-based materials, other good wave-absorbing materials have also attracted the attention of researchers. An et al. [133] prepared a flexible and recyclable SiC nanofibrous aerogel (SiCnf) using electrospinning and freeze-drying methods. The high aspect ratio of SiC nanofibers, internal defects, grain boundaries, and their synergistic effects contribute to the good electromagnetic wave absorption performance of SiCnf. Sun et al. [134] used polyimide nanofibers (PINFs) as three-dimensional assembly units and MXene as a functional filler to prepare three types of MXene/PINF aerogels through different freezing processes. As shown in Figure 11c, MXene/PINF-S exhibits the best performance with an RL_min_ of −37.9 dB and an EAB of 3.3 GHz.This is due to the fact that the pore size of MXene/PINF-S is minimized and the distribution of MXene nanosheets is improved, which facilitates electron migration, leading to enhanced conductive loss, further attenuating the electromagnetic wave energy and promoting the reflection and scattering of the electromagnetic wave (Figure 11d). Doping with elements or particles can affect the overall dielectric loss of the material, thereby influencing its electromagnetic wave absorption capability. Pan et al. [135] prepared a Ce^3+^-doped composite nanofibrous aerogel. The doping of Ce^3+^ affects the ferromagnetic resonance of the ferrite material, improving the microwave absorption capability with an EAB of up to 4.3 GHz, covering 93% of the C-band, and demonstrating good radar stealth capability.

However, there are still challenges to be addressed in the preparation and application of fibrous porous materials for radar stealth. Firstly, introducing multiple dielectric or magnetic loss materials during the material preparation process can complicate the material composition and increase costs, which is not conducive to serving as a base for radar stealth materials. Secondly, it is necessary to establish precise theoretical models that consider factors such as impedance matching, dielectric, and magnetic losses to guide the preparation and synthesis of wave-absorbing materials. Additionally, the current characterization of electromagnetic wave absorption properties focuses on an effective absorption bandwidth, with insufficient attention paid to low-frequency wave absorption capability. Good low-frequency wave absorption performance is crucial for targets to evade detection by decimeter and meter wave radars, making it more suitable for future battlefields. Finally, the wave-absorbing properties of materials in complex environments (high temperature, extreme cold) are also key to maintaining long-term radar stealth performance.

## 5. Conclusions and Prospects

This paper reviews the development of fibrous three-dimensional porous materials and classifies them into nanofibrous and microfibrous porous materials from the difference in fiber constituent units. Four common molding methods for fibrous three-dimensional porous materials are introduced as follows: vacuum forming, gel solidification, freeze-casting, and impregnation stacking, and the advantages and disadvantages of each method are summarized. In addition, the application prospects of fibrous three-dimensional porous materials in the stealth field are discussed. It can be seen that fibrous three-dimensional porous materials not only provide convenience for human beings in daily life and industrial production, but also improve the survivability of targets on the battlefield, which is an advanced intelligent material with excellent comprehensive performance. Although fibrous three-dimensional porous materials have made great progress, there are still many challenges from the design of materials to the actual application process, this paper puts forward the following outlook in view of these challenges, hoping to play a certain role in promoting fibrous three-dimensional porous materials.

1. At present, most fibrous three-dimensional materials are prepared by fixing the fiber skeleton with binders. The physical and chemical bonding of binders at the fiber overlap keeps the stability of the fiber matrix. However, the choice of different fiber monomers and binders has a great impact on the performance of the final material. Neither binders with low strength after sintering nor binders with an excessively high sintering temperature should be chosen. The former will affect the comprehensive mechanical properties of the material, while the latter will affect the characteristics of the fiber monomer itself. Meanwhile, the addition of binders will also affect the pore structure of the material. Therefore, the selection of binders, the amount of addition, and the precise control of the sintering temperature is a challenge. How to achieve the good structural stability of the material without binders is a key issue. Natural nanofibers, such as bacterial cellulose and cellulose nanofibers, are rich in surface functional groups. They can form stable self-supporting structures through physical bonding and chemical cross-linking without additional binders. With a more excellent compression resilience, they are good choices for high-performance fibrous porous materials. Inspired by this, surface grafting modification and other processes can be performed on synthetic high-modulus fibers to endow them with self-cross-linking ability, thus obtaining fibrous porous materials with both a high compression resilience and high strength. This is an important trend for future development.
2. Although the unique porous structure of fibrous three-dimensional porous materials can weaken the transmission of thermal energy, sound energy, and microwaves, the underlying mechanism is still immature. While researchers have utilized simulation methods to demonstrate the impact of structure on performance, the established models do not fully align with the actual internal structure, and the simulation methods are limited. Therefore, they cannot fully simulate the actual transmission behavior of thermal energy, sound energy, and microwaves within the material. Consequently, it is crucial to establish a transmission theory for thermal energy, sound energy, microwaves, and other energies within fibrous porous materials, and to construct a theoretical model that incorporates the effects of various properties of fibrous porous materials on the transmission processes of these energies. However, due to the limitations of modeling software and the complexity of the internal structure of fibrous porous materials, establishing an accurate theoretical model remains a significant challenge. In the future, a combination of extensive theoretical simulations and practical applications will be necessary to continuously improve and optimize the model. Only by utilizing an accurate theoretical model to guide the design of fibrous porous materials can we achieve the optimal material selection for different application scenarios, better respond to extreme conditions, and reduce production costs.
3. In the design process of fibrous three-dimensional porous materials, more attention is paid to their compression resilience. Indeed, the demand for material compression resilience is more extensive in practical application scenarios. However, in some specific scenarios, other properties such as bending resistance and stretchability are of greater concern, especially when used as stealth coating materials, where bending and stretching characteristics are prioritized. Nonetheless, the stretching and bending of materials often involve stress in multiple directions, while compression mainly involves unidirectional stress. Moreover, stretching and bending are more likely to lead to material damage or significant deformation, posing a major challenge to improving the comprehensive mechanical properties of fibrous three-dimensional porous materials. In the future, the three-dimensional structure of fibrous porous materials can be improved through various methods, such as changing the internal fiber orientation and constructing negative Poisson’s ratio structures with specific mechanical properties. By altering the stress structure and force transmission mode of the materials, fibrous three-dimensional porous materials with excellent impact resistance, shear resistance, and fracture resistance can be produced.
4. The current detection technology tends to be diversified, and a single means of stealth cannot be effectively camouflaged, so the development of multifunctional stealth materials has become the focus of research. However, stealth materials with different functions may mean the introduction of various components, which would influence the overall weight and cost of the materials, and how to enable the materials to possess multifunctional stealth properties within limited installation space is the greatest challenge in the process of design and production. The structural and functional modification of fibrous porous materials is a good choice to solve this problem, and the future direction of development is to design both a variety of functional characteristics of fibrous porous materials, which can be prepared through the regulation of the pore structure of heat-insulating–acoustic absorbing integration of materials for infrared and acoustic stealth, through the selection of the substrate to prepare the heat-insulating–wave absorption integration of materials for infrared and radar stealth, or to simultaneously modulate the pore structure of the material, change the matrix, and give the fiber surface functionalization characteristics, so as to prepare heat-insulating–acoustic and wave-absorbing integrated materials for infrared, acoustic, radar stealth, etc.. In addition, micro-nanofiber composite aerogels can be prepared in order to expand the range of its pore structure and extend the space of its functionalization modification.
5. Currently, the preparation of fibrous porous materials is mostly confined to the laboratory stage, the materials produced are mainly small-sized samples, and the batch production of large-sized samples remains unresolved. The main reasons lie in the issues of cost and usability. On the one hand, the preparation of large-sized samples requires more fibers and solvents, as well as larger drying equipment and a longer drying time, which determines higher costs and technological difficulties. On the other hand, it remains a significant challenge whether large-sized samples can maintain and extend the mechanical properties of small-sized samples. As the radial size of the samples increases, their deflection may not be able to support their own gravity and cause fracture. Therefore, more attention needs to be paid to various mechanical performance indicators of the material during the design of large-sized samples to obtain practically usable large-sized fibrous porous materials. As a result, searching for low-cost, green, and renewable fiber raw materials, and conducting in-depth research on the relationship between material mechanical properties and size, are inevitable paths for fibrous three-dimensional porous materials to move towards practical applications.

In summary, fibrous three-dimensional porous materials still face many challenges. However, with the deepening of research and exploration, fibrous three-dimensional porous materials will definitely achieve new leaps from structure to function, from preparation to application, and from theory to practice. In the near future, we will see their presence in various industries.
